# Beyond chronological age: uncovering disease-dependent variability in AI-driven pediatric bone age assessment

**DOI:** 10.1186/s13244-026-02477-0

**Published:** 2026-11-14

**Authors:** Felix Schön, Christoph Meißner, Stephanie Spieth, Kathleen Pätzold, Sophia Freya Ulrike Blum, Ralf-Thorsten Hoffmann, Jens-Peter Kühn, Marie-Luise Kromrey

**Affiliations:** Institute and Polyclinic for Diagnostic and Interventional Radiology, Faculty of Medicine and University Hospital Carl Gustav Carus Dresden, TU Dresden, Dresden, Germany

**Keywords:** Artificial intelligence, Bone age, Greulich and Pyle, Hand, Pediatric

## Abstract

**Objectives:**

To assess performance and agreement of a commercial artificial intelligence (AI) tool relative to routine pediatric radiologists’ bone age assessments in a large pediatric cohort, with focus on different groups of growth disorders.

**Materials and methods:**

One thousand five hundred sixteen anterior-posterior hand radiographs of pediatric patients (50.6% boys, mean age 10.84 ± 3.66 years) were retrospectively analyzed using the AI tool BoneAge. AI results were compared with board-certified pediatric radiologists’ reports using Bland–Altman plots, Spearman correlations, and the intraclass correlation coefficient (ICC). Subgroup analyses were performed for three major indications: genetic/neuroendocrine (*n* = 1179), orthopedic (*n* = 152), and oncologic disorders (*n* = 185).

**Results:**

Mean absolute difference (MAD) in estimated bone age by radiologists and AI was 6.4 ± 7.0 months for the whole cohort, with complete concordance in 45.1% of cases. Best results were achieved in orthopedic questions with an MAD of 4.1 ± 5.6 months (genetic/neuroendocrine: 6.6 ± 7.2, oncologic: 6.7 ± 6.3 months). There was significant correlation between AI and radiologists for the whole study population (ρ = 0.980), and the three main groups: genetic/neuroendocrine (ρ = 0.974), orthopedic (ρ = 0.952), and oncologic indications (ρ = 0.988). ICCs for the entire cohort and the main indication groups were > 0.9, indicating excellent agreement between radiologist and AI.

**Conclusion:**

The investigated algorithm showed good agreement with bone age assessments performed by pediatric radiologists in a large pediatric cohort. Novel to previous studies, the indication for imaging was considered, demonstrating that agreement between algorithm output and radiologists’ assessments varied across disease groups, with highest agreement observed in orthopedic indications.

**Key Points:**

***Question*** The performance of artificial intelligence tools for bone age assessment among different subgroups of pediatric patients according to underlying diseases has not yet been investigated.

***Findings*** Automated bone age assessment reliably correlates with estimation by pediatric radiologists. Software output differs according to disease groups, with best results for orthopedic indications.

***Critical relevance statement*** In a real-world scenario, the commercial software showed good agreement with routine bone age assessments in a large cohort of pediatric patients. The observed disease-dependent variation suggests that radiologists should consider disease-specific differences when using additional automated tools.

**Graphical Abstract:**

**Figure:**
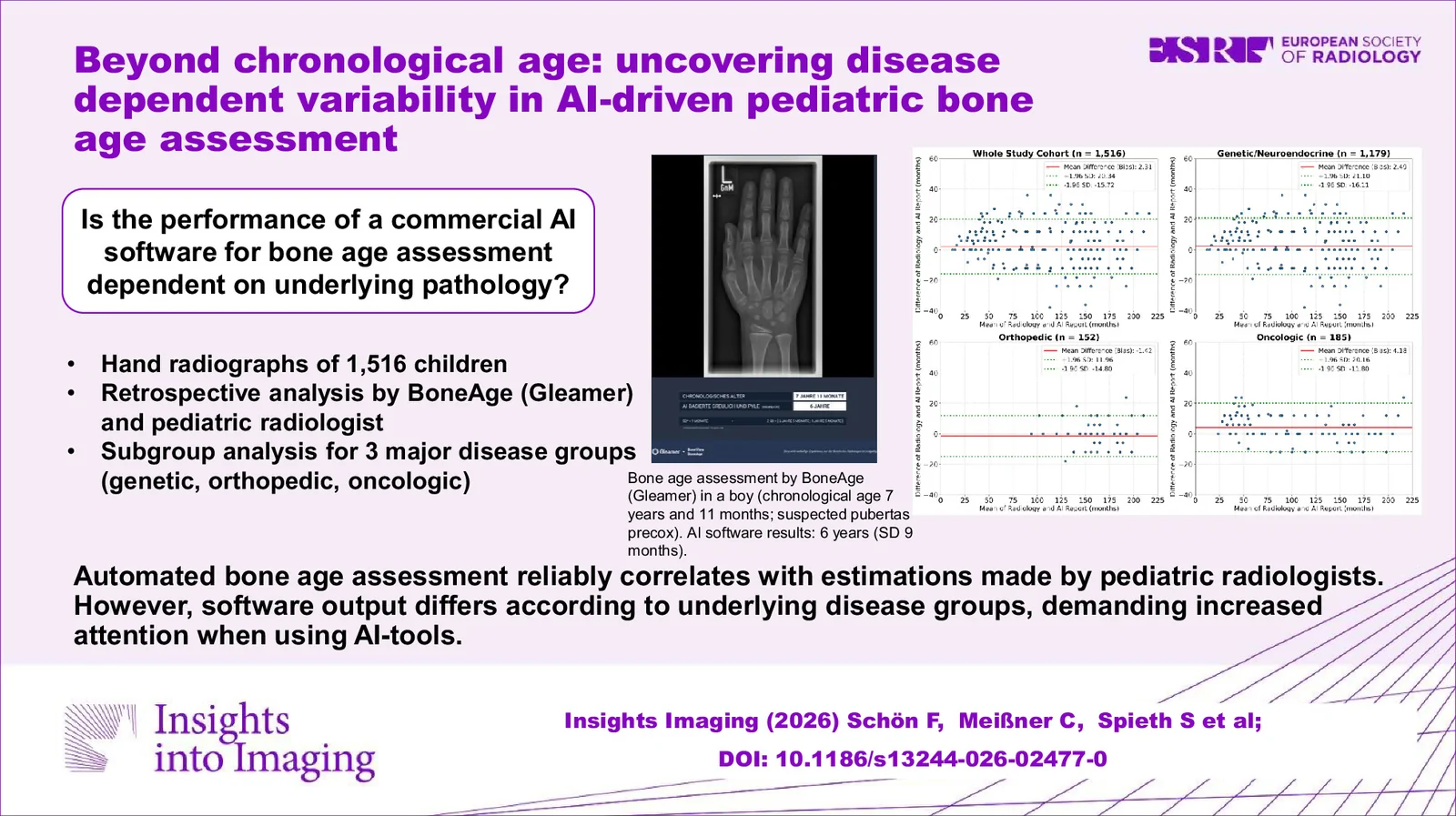

## Introduction

Skeletal maturity in children is influenced by several external and internal factors, such as social surroundings, nutrition, metabolism and genetics, disease, or endocrine dysfunction [1]. The assessment of bone age in children, defined as skeletal maturity relative to chronological age, is a routine task in pediatric medical disciplines, especially in endocrinology and orthopedics, to determine a child’s developmental status, including growth potential, monitoring of treatment response, or forensic purposes [2]. Here, the manual Greulich and Pyle (G&P) method constitutes the most commonly deployed technique, with 76% of radiologists and endocrinologists preferring it over other procedures [3]. Established on a reference of Caucasian middle-class children in the USA in the 1930s and early 1940s, X-ray images of the left and/or right hand are compared with reference images in the G&P atlas, first published in 1959, which depict the typical sequence of ossification in the hand and wrist over time [1, 4]. Although the G&P method is easily implemented, it suffers from known inter- and intraobserver inconsistencies, and requires experienced readers and time to perform [3, 5–8].

Against the background of increasing workload in radiology departments and the growing need for standardized assessments, the role of artificial intelligence (AI) in medical imaging has become increasingly present in recent years, with several commercially available tools already existing on the market, for instance, for lesion detection and characterization, image quality improvement or in the field of musculoskeletal radiology for fracture detection [9]. AI-based methods for automated bone age determination have gained substantial interest, not only to avoid this tedious task. Several studies have reported that AI-based assessments may achieve performance comparable to that of experienced radiologists, potentially reducing reporting time while maintaining diagnostic reliability [2, 10–15].

In addition to overall performance, it is crucial to understand whether AI-based bone age assessment maintains its accuracy across different clinical subgroups commonly encountered in pediatric radiology. Children with endocrine disorders, orthopedic conditions, or those undergoing oncological treatment may present with growth patterns that differ from those in healthy children, potentially affecting the accuracy of both manual and automated bone age assessment. To our knowledge, this aspect has not been investigated before, neither using manual G&P nor AI-based bone age assessment.

Therefore, this study aims to evaluate the agreement of a commercially available AI-based bone age assessment tool in comparison to the manual G&P assessment by pediatric radiologists, analyzing accuracy and consistency in a large clinical cohort of pediatric patients with particular emphasis on the performance of the AI system among different subgroups of pediatric patients with endocrinological, orthopedic, and oncological conditions. Our findings seek to provide evidence on the potential role of AI in routine bone age assessment and its integration into pediatric radiology workflows.

## Material and methods

### Study design

The retrospective study was approved by the ethics committee of the University Hospital Carl Gustav Carus, TU Dresden (BO-EK-191052024) and conforms to the Declaration of Helsinki in its present form. Written consent was waived due to the retrospective study design.

**Fig. 1:**
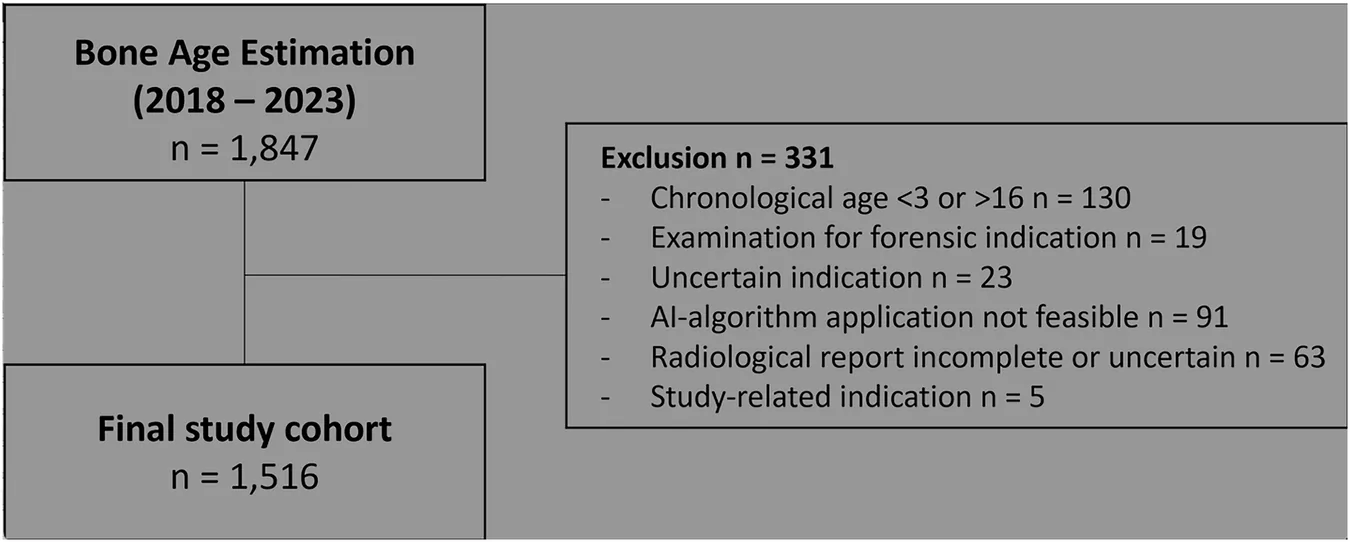
Application of exclusion criteria on the study cohort

Between 2018 and 2023, a total of 1847 pediatric patients underwent radiographic bone age assessment of the hand and wrist at our department in a tertiary care center. Three hundred thirty-one patients were excluded according to the following criteria (Fig. 1): (1) chronological age < 3 or > 16 years (according to the approved age range of the AI software; see description below), (2) examinations for forensic indications, 3) uncertain indication for imaging, (4) application of the AI algorithm was not feasible, (5) incomplete or uncertain radiological report, and (6) examination as part of a clinical trial. The final study cohort encompassed 1516 children.

This study was conducted independently of the software provider Gleamer, which was not involved in any aspect of the study design, data interpretation, or manuscript development.

### Data acquisition

#### X-ray parameters

All pediatric patients underwent a standardized anterior-posterior projection X-ray examination of the left hand (in only one patient, the right hand was examined), using the Luminos dRF Max system (Siemens Healthineers) without a scatter grid or additional filter. Acquisition parameters were as follows: tube voltage 55 kV, tube current 0.82 mAs.

#### Image and data analysis

Demographic data (chronological age and gender), as well as clinical information on underlying diseases and developmental disorders, were obtained for all patients from the in-house medical database. Indications for radiological bone age estimation are summarized in Table 1 and were subdivided into three main groups (genetic/neuroendocrine vs orthopedic vs oncologic), as well as 13 more specific subgroups (1 somatomegaly, 2 microsomia, 3 pubertas precox, 4 pubertas tarda, 5 pre-chemotherapy/stem cell therapy, 6 post or ongoing chemotherapy/stem cell therapy, 7 scoliosis, 8 leg length discrepancy, 9 congenital adrenal hyperplasia, 10 other systemic diseases/bone disorders, 11 others, 12 growth assessment without underlying pathology, 13 focal orthopedic conditions). Subgroups 10 and 11 unite cases with small patient numbers, which therefore could belong to different main groups; for instance, rachitis or hypophosphatemic rickets were assigned to the genetic/neuroendocrine main group, whereas chronic recurrent multifocal osteomyelitis was assigned to the orthopedic group. Other entities were: GLASS syndrome, osteochondrodysplasia, pseudohyperparathyroidism, osteopetrosis, osteogenesis imperfecta, or suspected bone metabolism disorder.

**Table 1:** Overview of clinical indications for radiographic bone age assessment

Main indications	Subgroups
Genetic/neuroendocrine	Somatomegaly (tall stature)
	Microsomia (short stature)
	Pubertas precox
	Pubertas tarda
	Congenital adrenal hyperplasia (CAH)
	Other systemic disorders (e.g., rachitis, hypophosphatemic rickets)
	Others
Orthopedic	Scoliosis
	Discrepancy in leg length
	Focal orthopedic conditions (e.g., genu valgum, hallux valgus, pes planovalgus)
	Growth assessment without underlying pathology
	Other systemic bone diseases (e.g., Chronic Recurrent Multifocal Osteomyelitis [CRMO])
Oncologic	Pre-chemotherapy/stem cell therapy
	Post- or ongoing chemotherapy/stem cell therapy
	Others

All X-ray images were provided with a written report including bone age estimation according to the G&P method by one of three in-house board-certified pediatric radiologists (with 3 years of subspecialty training in pediatric radiology and an overall experience between 10 and 30 years), with knowledge of the patient’s gender, chronological age, and pathology. During the reporting process, no assistance from AI algorithms was utilized, as no such systems were available at our institution throughout the period from 2018 to 2023. The radiological reports served as the reference standard.

#### AI algorithm

**Fig. 2:**
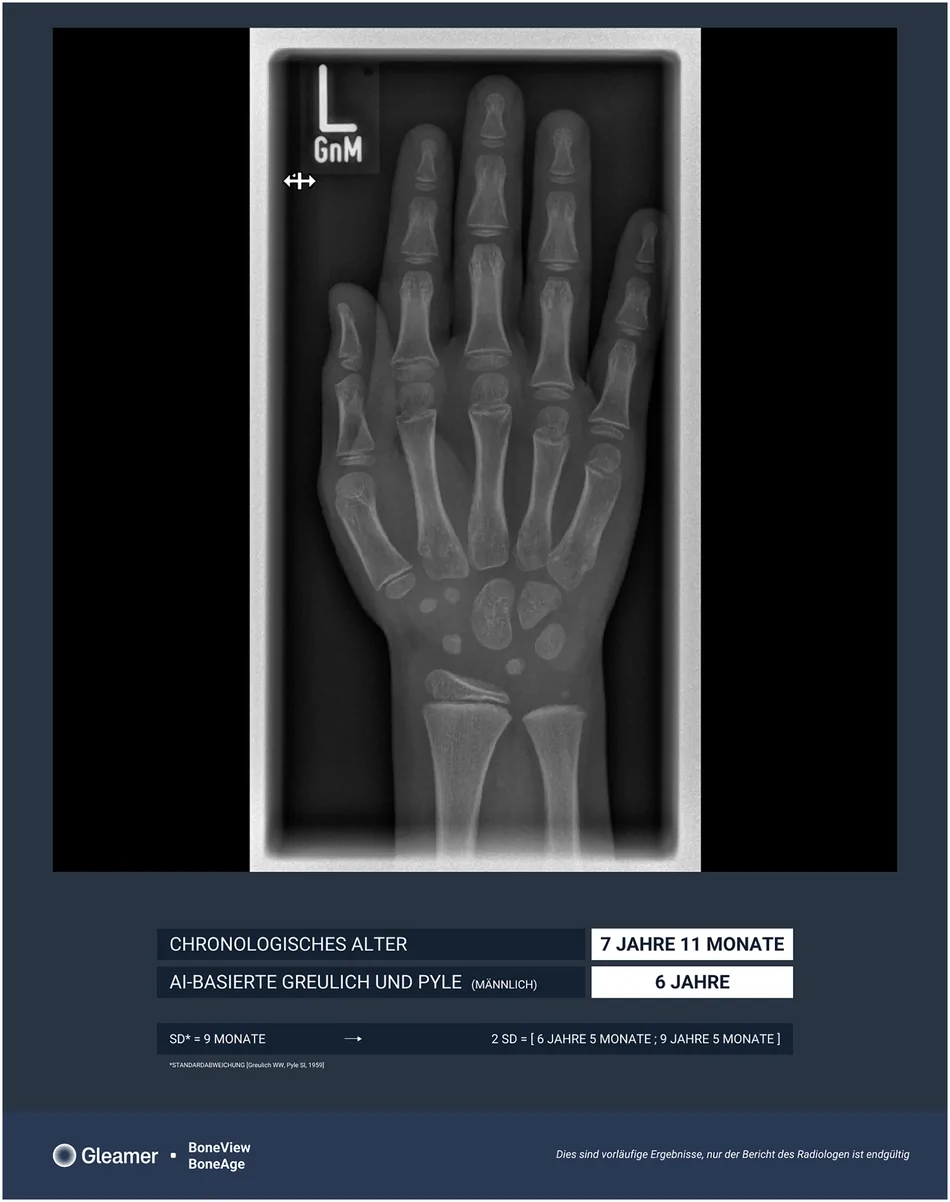
Example of a radiograph of the left hand and wrist after automated bone age assessment by the software BoneAge (Gleamer) in a young boy with chronological age of 7 years and 11 months (indication for imaging: suspected precocious puberty). The bone age estimation by a pediatric radiologist was 6 years, by AI software, 6 years (SD 9 months)

The commercial software BoneAge version 2.6.2 (Gleamer), since March 2023 a CE-certified tool based on the G&P Atlas of Skeletal Development of the Hand and Wrist [4], was retrospectively applied to all examinations enrolled in this study. X-ray images in DICOM format are processed by the software through a cloud server by the AI company. The tool uses a deep learning algorithm, model training and validation have been described previously [2]. Results are sent to the department’s picture archiving and communication system (PACS) as an annotated DICOM image (Fig. 2).

BoneAge rejects analysis if the chronological age is below 3 years. In accordance with the recommendation of the European Society of Pediatric Radiology [16], the software is not designed for forensic use, meaning it cannot determine whether a person is legally an adult, and therefore sets an upper limit of ≥ 17 years.

#### Statistics

Categorical variables were presented as absolute and relative frequencies, continuous variables as mean and standard deviation (SD). Normal distribution was tested using the Kolmogorov-Smirnov test.

Baseline characteristics of all pediatric patients were compared across the main clinical indications using Kruskal–Wallis and Chi-squared tests. Wilcoxon signed-rank tests were applied to compare radiological and AI-based bone age estimates across all indications. Mean differences and mean absolute differences (MAD) between the radiological and AI-based assessments were calculated. In addition, the number of concordant findings was recorded, as well as the number of discrepancies within a maximum deviation of six months. To assess differences in bone age estimation between different genders, the Mann–Whitney *U*-test was used. To further evaluate the agreement between radiologists and AI-based bone age estimations, intraclass correlation coefficients (ICC) were calculated. Bland–Altman plots were generated to graphically assess the level of agreement between radiologists and the AI-based algorithm.

To assess correlation between radiologists’ and AI-derived reports, Spearman correlations were performed. Subsequently, differences were correlated with patients’ chronological age, again using Spearman correlations.

The significance level was set at α < 0.05. Statistical analyses were performed with SPSS (Version 30; IBM) and Python (Version 3.13.5, https://www.python.org/).

## Results

A total of 1516 hand X-rays were analyzed retrospectively. The mean chronological age ± SD of the entire cohort was 10.84 ± 3.66 years, with 767 male (50.6%) and 749 female (49.4%) patients. Demographic data of the entire patient cohort and subgroups can be found in Table 2.

**Table 2:** Demographic data according to three main and 13 specific subgroups

Indications	Patients, *n*	Chronologic age (years, mean ± SD)	Male/female. n
Genetic/neuroendocrine	1179 (77.8%)	10.71 ± 3.49	605 (51.3%)/574 (48.7%)
Somatomegaly	206 (13.6%)	10.97 ± 3.05	112 (54.4%)/94 (45.6%)
Microsomia	655 (43.2%)	11.04 ± 3.52	407 (62.1%)/248 (37.9%)
Pubertas precox	176 (11.6%)	8.15 ± 2.05	17 (9.7%)/159 (90.3%)
Pubertas tarda	74 (4.9%)	13.71 ± 2.78	35 (47.3%)/39 (52.7%)
Congenital adrenal hyperplasia (CAH)	37 (2.4%)	9.21 ± 3.54	19 (51.4%)/18 (48.6%)
Orthopedic	152 (10.0%)	13.86 ± 1.95	56 (36.8%)/96 (63.2%)
Scoliosis	81 (5.3%)	14.62 ± 1.61	17 (21.0%)/64 (79.0%)
Discrepancy in leg length	27 (1.8%)	13.55 ± 1.42	14 (51.9%)/13 (48.1%)
Focal orthopedic conditions	31 (2.0%)	12.97 ± 2.14	15 (48.4%)/16 (51.6%)
Growth assessment without underlying pathology	9 (0.6%)	13.54 ± 1.77	9 (100%)/0 (0%)
Oncologic	185 (12.2%)	9.17 ± 4.41	106 (57.3%)/79 (42.7%)
Pre-chemotherapy/stem cell therapy	110 (7.3%)	8.66 ± 4.53	60 (54.5%)/50 (45.4%)
Post or ongoing chemotherapy/stem cell therapy	54 (3.6%)	9.38 ± 4.25	35 (64.8%)/19 (35.2%)
Other systemic disorders/bone diseases	39 (2.6%)	10.93 ± 3.52	20 (51.3%)/ 19 (48.7%)
Others	17 (1.1%)	11.04 ± 4.66	7 (41.2%)/10 (58.8%)

Cases in the last two subgroups, other systemic disorders/bone diseases and others*,* could be assigned to more than one main indication and were allocated to the most appropriate main group based on clinical relevance

*SD* standard deviation

Mean estimated bone age by pediatric radiologists was 10.86 ± 3.86 years, the AI software delivered a mean bone age of 10.67 ± 4.08 years, resulting in a mean difference of 2.31 ± 9.20 months and an MAD of 6.38 ± 7.01 months, which was statistically significant (*p* < 0.001). In 45.1% (684 cases), radiologists and AI software delivered concordant results. When allowing a maximum age estimation error of 6 months, readers and software agreed in 60.2% (912 cases). There was a significant difference in bone age reported by radiologists and AI regarding gender: For the whole study group, the mean difference between the two methods was 0.69 ± 8.95 months for girls and 3.89 ± 9.17 months for boys (*p* < 0.001). The MAD was 5.86 ± 6.80 months for girls and 6.89 ± 7.19 months for boys (*p* = 0.013).

### Agreement in the main disease groups

According to the clinical question, 1179 (mean age 10.71 ± 3.49 years, 51.3% boys) of the entire 1516 children included were distributed into the genetic/neuroendocrine group, 152 (13.86 ± 1.95 years, 36.8% boys) belonged to the orthopedic group, and 185 (9.17 ± 4.41 years, 57.3% boys) to the oncologic group.

Similar to the entire study cohort, radiologists’ and AI’s mean age estimation significantly differed in all main disease groups: In cases of a genetic/neuroendocrine indication, the pediatric radiologists documented a mean bone age of 10.68 ± 3.60 years, and the AI software 10.47 ± 3.79 years, resulting in an agreement rate of 43.8%, a mean difference in bone age estimation of 2.49 ± 9.49 months (*p* < 0.001) and an MAD of 6.63 ± 7.23 months. The oncologic group yielded a mean estimated bone age by radiologists of 9.15 ± 4.76 years, whereas AI reported a value of 8.80 ± 5.04 years, showing agreement in only 40.5% of cases, a mean difference of 4.18 ± 8.15 months (*p* < 0.001) and an MAD of 6.65 ± 6.30 (*p* < 0.001). Best results were documented for orthopedic indications, where mean bone age assessed by radiologists was 14.33 ± 2.08 years, and by the software 14.45 ± 2.12 years (*p* = 0.016, 61.2% agreement), equaling a mean difference of −1.42 ± 6.83 months and an MAD of 4.11 ± 5.63 months. When allowing a divergence of 6 months in age estimation, software and human readers matched in 59.5% in the genetic/neuroendocrine group, 54.0% in the oncologic group, and 73.0% in the orthopedic group.

**Fig. 3:**
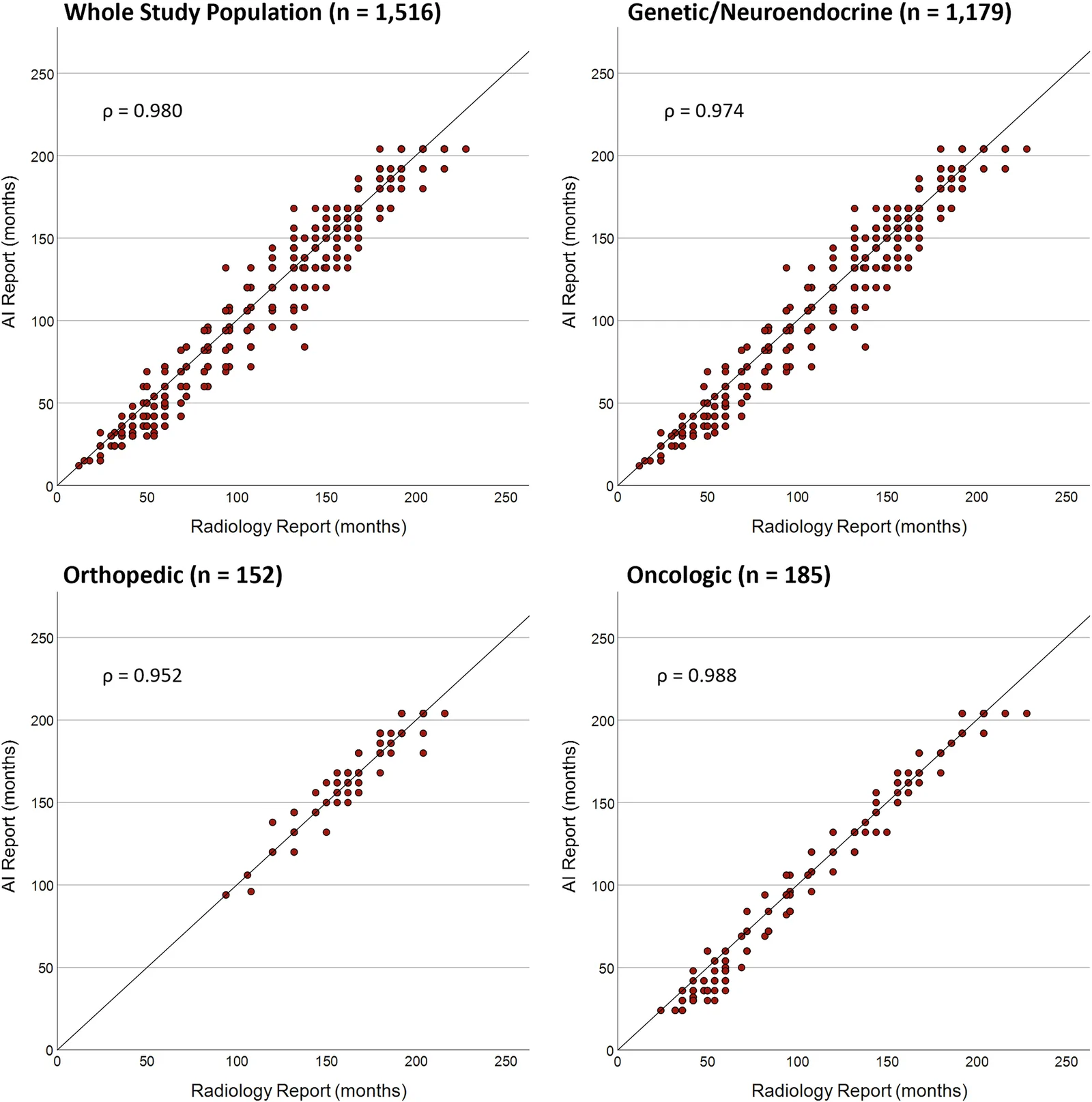
Spearman correlation analysis for bone age estimation made by a radiologist (reference standard) against the AI algorithm showed a strong and significant correlation for the entire study cohort, as well as the three main indication groups: genetic/neuroendocrine, orthopedic, and oncologic indications, with high Spearman-ρ values

There was a strong and significant correlation between the software and radiologists (serving as the reference standard) regarding bone age estimation for the whole study population (ρ = 0.980, *p* < 0.001), as well as the three main groups: genetic/neuroendocrine (ρ = 0.974, *p* < 0.001), orthopedic (ρ = 0.952, *p* < 0.01), and oncologic indications (ρ = 0.988, *p* < 0.001) (Fig. 3).

**Fig. 4:**
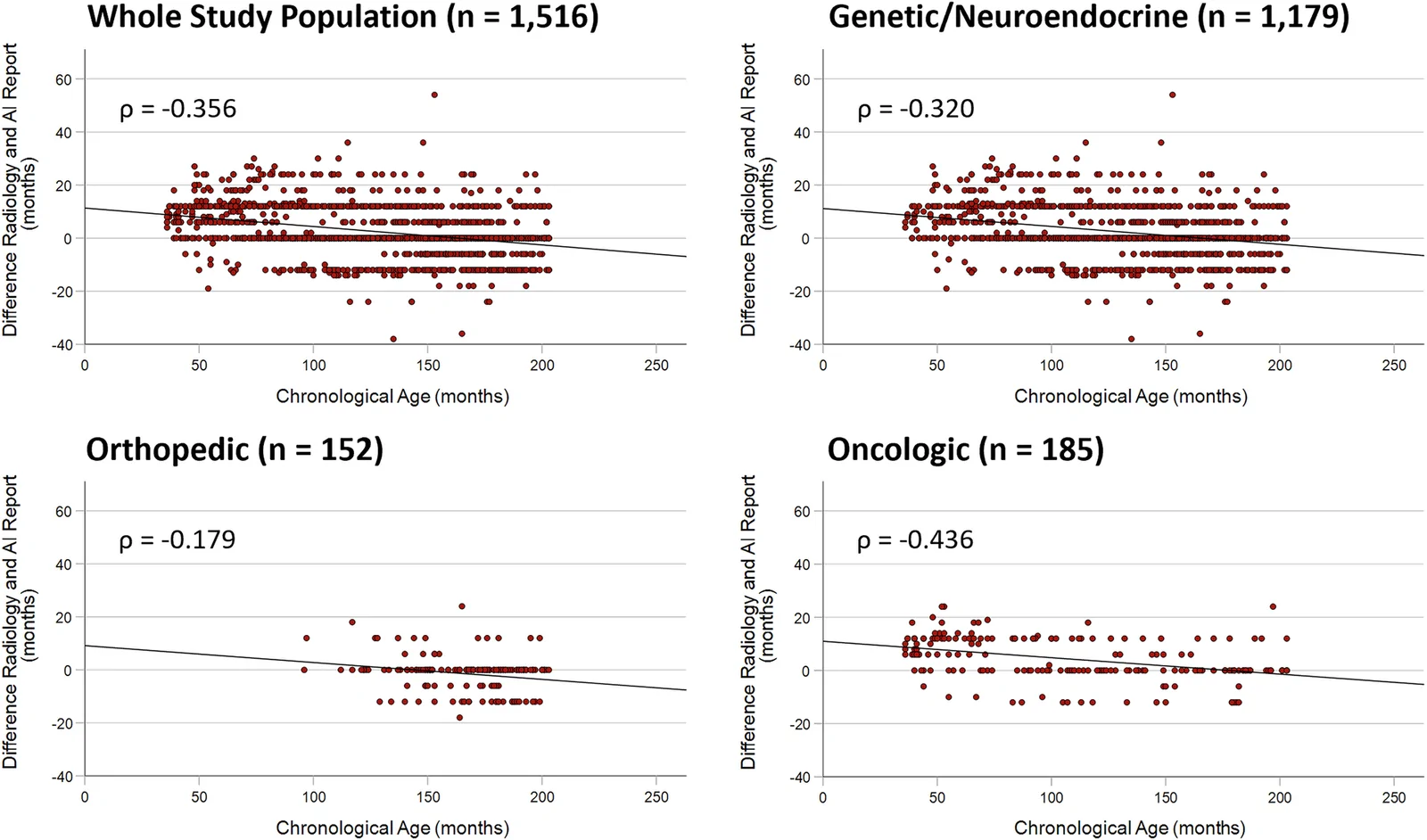
Spearman correlation analysis for chronological age against the difference in bone age estimation made by radiologists and the AI algorithm showed a negative correlation for the entire study cohort, as well as the three main indication groups, with modest Spearman-ρ values

Statistical analysis further demonstrated a significant, modestly negative correlation between chronological age and the difference between radiologist-estimated and AI-derived bone age for the whole population (ρ = −0.356, *p* < 0.001), the genetic/neuroendocrine group (ρ = −0.320, *p* < 0.001), orthopedic group (ρ = −0.179, *p* = 0.028), and the oncologic group (ρ = −0.436, *p* < 0.001) (Fig. 4): The older the child, the smaller the difference in age estimation of AI algorithm and radiologist becomes.

Concerning gender, significant differences in bone age estimation between radiologists and AI software were seen in the genetic/neuroendocrine group (mean difference girls: 0.86 ± 9.21 months, boys: 4.04 ± 9.50 months; *p* < 0.001), but not in the orthopedic (mean difference girls: −2.25 ± 6.23 months, boys: 0.00 ± 7.69 months; *p* = 0.053) or oncologic group (mean difference girls: 3.04 ± 9.01 months, boys: 5.04 ± 7.38 months; *p* < 0.244).

### Subgroup analyses

Regarding the 13 specific subgroups, no significant differences in bone age estimation between radiologists and AI software were shown for the indications pubertas tarda (*p* = 0.841), leg length difference (*p* = 0.067), focal orthopedic conditions (*p* = 0.582), other systemic bone diseases/disorders (*p* = 0.301), and the very heterogeneous subgroup of other not directly referable indications (*p* = 0.271). All subgroup parameters are summarized in Table 3.

**Table 3:** Specific subgroup analysis

Subgroup	Bone age radiologist (years, mean age ± SD)	Bone age AI (years, mean age ± SD)	Complete agreement	Agreement with max. 6 months aberration	*p*-value
Somatomegaly	11.89 ± 2.91	11.78 ± 3.01	91 (44.2%)	135 (65.5%)	**0.035**
Microsomia	10.37 ± 3.83	10.14 ± 4.06	272 (41.5%)	382 (58.3%)	**< 0.001**
Pubertas precox	9.44 ± 2.53	9.12 ± 2.60	93 (52.8%)	102 (58.0%)	**< 0.001**
Pubertas tarda	12.94 ± 2.80	12.94 ± 2.87	34 (45.9%)	46 (62.2%)	0.841
CAH	11.00 ± 3.78	10.41 ± 3.86	10 (27%)	14 (37.8%)	**0.005**
Scoliosis	15.18 ± 1.59	15.35 ± 1.53	51 (63.0%)	57 (70.4%)	**0.016**
Discrepancy in leg length	14.00 ± 1.29	14.22 ± 1.47	14 (51.9%)	20 (74.1%)	0.067
Focal orthopedic conditions	13.23 ± 2.59	13.29 ± 2.52	20 (64.5%)	23 (74.2%)	0.582
Growth assessment without underlying pathology	14.56 ± 1.90	14.22 ± 1.72	4 (44.4%)	8 (88.9%)	**0.034**
Pre-chemotherapy/stem cell therapy	8.65 ± 4.91	8.35 ± 5.16	45 (40.9%)	59 (53.6%)	**< 0.001**
Post- or ongoing chemotherapy/stem cell therapy	9.24 ± 4.57	8.80 ± 4.92	19 (35.2%)	27 (50.0%)	**< 0.001**
Other systemic disorders/bone diseases	10.41 ± 4.03	10.47 ± 4.22	21 (53.8%)	28 (71.8)	0.301
Others	10.86 ± 4.81	10.63 ± 5.22	10 (58.8%)	11 (64.7%)	0.271

Wilcoxon signed-rank test. Statistically significant values are presented in bold

*AI* artificial intelligence, *CAH* congenital adrenal hyperplasia, *SD* standard deviation

### Intraclass correlation

**Fig. 5:**
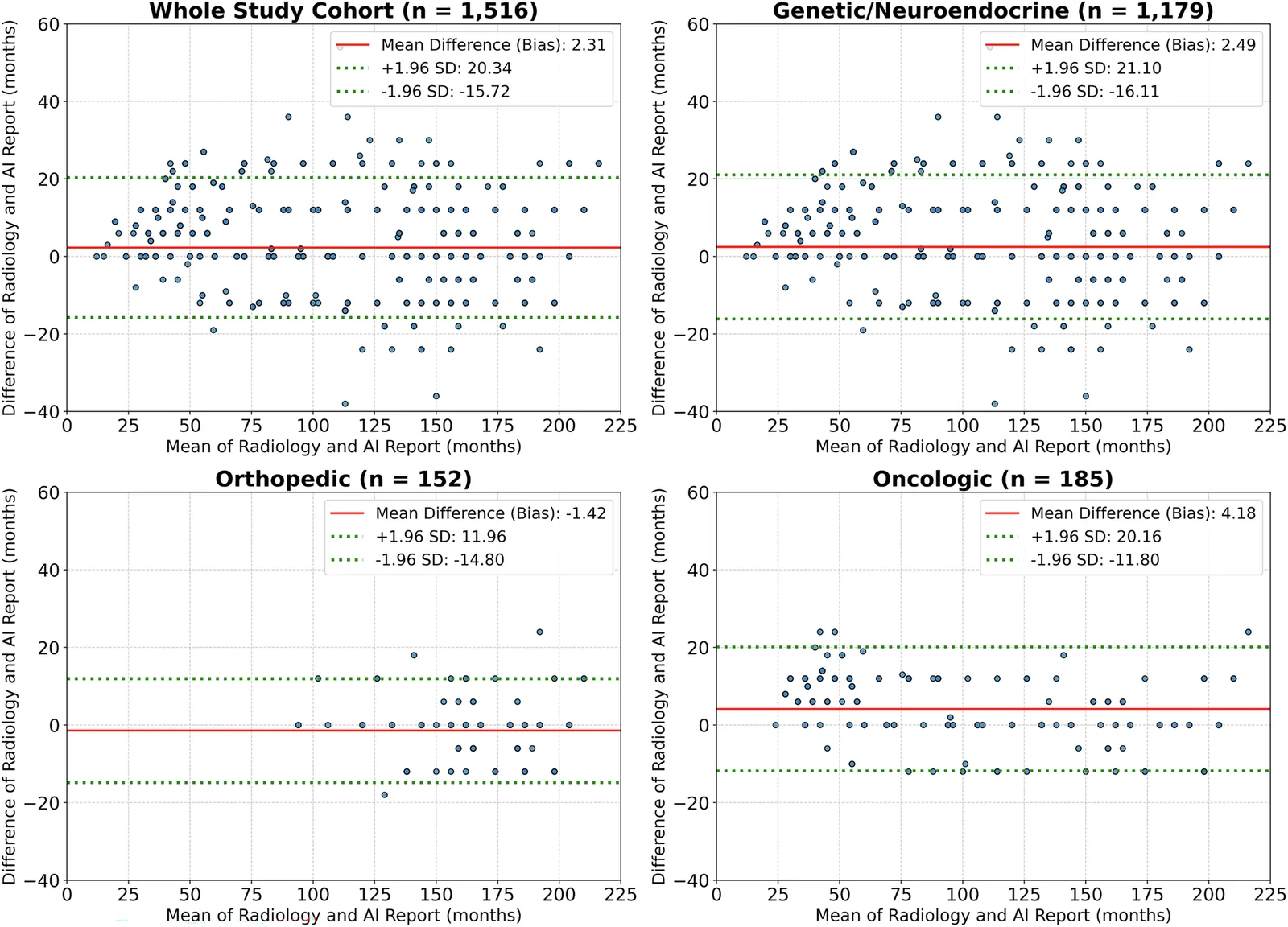
Bland–Altman plots demonstrate excellent agreement with corresponding ICC values > 0.9 between radiologist and AI-based bone age estimation for the entire cohort, as well as the three main indication groups genetic/neuroendocrine (B), orthopedic, and oncologic. Continuous line: mean difference; dashed lines: upper and lower limits of agreement

To further assess variability between radiologists and AI-based assessment of bone age, the ICC was calculated. The ICC for the entire cohort, the main indication groups, as well as among all 13 subgroups was > 0.9, indicating excellent agreement. The level of agreement for the whole study population and the three superordinate groups is depicted as Bland–Altman Plots in Fig. 5. Bland–Altman analysis showed small bias with a mean difference of 2.31 months (±9.20) for the entire cohort, 2.49 months (±9.49) for genetic/neuroendocrine, and −1.42 months (±6.83) for orthopedic indications. The highest differences were again observed for oncologic indications (4.18 months ± 8.15). Bland–Altman analyses for the 11 subgroups definitely attributable to one of the three main indication groups are provided as supplemental material (Suppl. 1).

## Discussion

In this retrospective study, we evaluated the agreement between a commercially available AI- tool and pediatric radiologists in terms of bone age assessment in a large cohort of pediatric patients, focusing on the performance in different disease and indication subgroups. We found that bone age estimated by the AI software was reliable, showing significant correlation with assessments by pediatric radiologists for the whole study group, as well as the three main indication groups. Across disease subgroups, the highest concordance between radiologists and AI was observed in orthopedic indications.

For a vast number of clinical questions in pediatrics, but also for forensic purposes, assessing bone age in children is inevitable. The most widely used G&P method, is—like any manual method carried out by human readers—highly dependent on the experience of the reader. As modifications in the course of bone growth may be subtle, the method is subject to substantial interobserver variability [8, 17]. In recent years, medicine has seen a trend towards the use of AI to revolutionize diagnostic imaging, risk stratification, and therapeutic decision-making in all fields of application. Assessing bone age in children by interpreting X-ray images of the hand is by far not the only, but a very good example of how an automated approach might present an exercisable alternative to manual assessment by providing rapid and, most notably, standardized and reproducible results. In particular, as the imaging process is typically highly standardized, bone age assessment represents a reliable example of AI application in radiology. The value of AI clearly lies in aiding radiologists in bone age assessment and accelerating the interpretation process. Moreover, it might be particularly beneficial for general radiologists or radiologists in training.

The performance of the AI algorithm examined in our study (BoneAge, Gleamer) was previously investigated by Nguyen et al [2]. The authors showed that the software performed significantly better than general radiologists in terms of MAD, correlation, and bias in predicting bone age. BoneAge also exhibited a higher sensitivity in a shorter timeframe than general radiologists. Other study groups working with different but similar AI algorithms (commercial software, such as BoneXpert (Visiana) or IB Lab PANDA (IB Lab GmbH), as well as newly designed algorithms) have likewise reported a significantly lower MAD in bone age assessment compared to general radiologists [12, 18, 19].

In our study, the MAD for the general study population of 6.38 months was slightly higher compared to the results reported by Nguyen et al (5.9 months), Gräfe et al (6.0 months), and a recent study in a Portuguese pediatric cohort using BoneAge (4.9 months) [2, 19, 20]. It should be noted that these differences between AI software and pediatric radiologists observed in our study were statistically significant—a result neglected in all previous studies, where the primary aim was often to compare AI with general radiologists or other clinicians, using pediatric radiologists as the reference standard. In general, most study groups considered AI software solutions comparable to expert readers in terms of bone age assessment. In our opinion, significant differences in bone age estimation between expert readers and AI, as shown in our study (and most likely also present in other studies, though not documented), have to be acknowledged when evaluating the performance of commercial AI tools. Nonetheless, the strong correlation, concordant with previous studies [2, 19, 21], and the very good ICCs observed in our cohort indicate a high overall agreement between AI-based and radiologist-based bone age assessment. However, these metrics do not imply full clinical interchangeability, as exact agreement was achieved in only 45.1% of cases and agreement within 6 months in 60.2%, indicating relevant case-level discrepancies. The 6-month threshold was selected as a pragmatic benchmark to compare agreement of radiologists with AI-based bone age assessment. However, the clinical relevance of such deviations is likely disease- and age-dependent, as a 6-month discrepancy may have greater implications in younger children than in adolescents. Overall, 6 months represent a balanced threshold that is sufficiently strict to identify clinically meaningful discrepancies while remaining robust to the degree of variability commonly encountered in bone age assessment. While our analysis focused on a clear comparison between AI and radiologists, the observed results suggest that the evaluated commercial AI software may serve as a supportive tool in aiding radiologists in bone age estimation, particularly since the clinical relevance of a mean absolute deviation of weeks to a few months remains uncertain, especially against the background of a certain natural variability.

Mean differences in bone age estimation between AI and radiologists were higher for boys compared to girls, for the entire cohort, as well as for the main indication groups, which supports previous findings by Simoes et al [20] and Gräfe et al [19], whereas Nguyen et al found no significant gender specific difference [2]. We further noticed that the extent of differences in months decreased with increasing patients’ age.

A clear strength of our study is the unprecedentedly high number of children included in an unselected cohort. Interestingly, a strategic pursuit by many investigators is to enable a more balanced cohort by selecting a specific number of children for each age group—an approach we did not follow in our study. Nguyen et al [2], for instance, collected eight boys and girls from each age interval between 5 and 17 years, resulting in a smaller study population of 206 patients. The same goes for Pape et al and Gräfe et al with 306 children [19, 22]. Often the entire age range of the employed software was not covered (e.g., Nguyen et al included children aged 5–17 years), and, more notably, different indications for imaging were not considered when dividing patients into age groups. This, however, could have had an important impact on bone age assessment, especially in conditions such as growth disorders. Furthermore, there is a highly heterogeneous composition of study populations across different studies, which was not taken into account in the respective analyses. For instance, in the study by Booz et al [12], 42.5% of children were examined for underlying microsomia, which may have introduced a selection bias.

In fact, to date, no study has specifically investigated different indications for bone age imaging. Our study demonstrates that AI performance in bone age estimation depends on the underlying indication, respectively disease: Best results were achieved for orthopedic indications with a mean difference of only 1.42 months, whereas a mean difference of 2.49 months was observed for genetic/neuroendocrine indications and 4.18 months for oncologic indications. Likewise, certain more deduced subgroups, especially with orthopedic backgrounds, showed more concordant results between radiologists and AI. One possible explanation for the reduced correlation observed in oncologic patients may be therapy- and disease-induced alterations of skeletal maturation that deviate from developmental patterns represented in the AI training datasets. However, this hypothesis cannot be further investigated within the present study design. Radiologists may recognize these atypical maturation patterns in a clinical context, whereas the algorithm is optimized for normal growth and, therefore, more prone to error in pathologic conditions. In return, indications in the orthopedic group mainly include indications not associated with general maturation retardation, such as scoliosis, focal orthopedic conditions (e.g., genu valgum, pes planovalgus, etc.), or even growth assessment without underlying pathology, so that overall bone age is more likely to follow normal patterns. These findings suggest that AI software tools should never serve as a standalone diagnostic tool but should always be validated by experienced radiologists, and that the latter should pay attention when using additional AI software for bone age estimation depending on the underlying disease.

Our study has some limitations. First, the retrospective study design and single-center setting limit the generalizability of our findings, for instance with regard to other ethnicities. Likewise, only one single commercial AI platform was evaluated—additional multicenter studies involving different AI systems are necessary to allow generalizability to a broader clinical practice. Second, pediatric radiologists used the G&P method to assess bone age with its sufficiently known limitations. Third, X-rays were analyzed by three pediatric radiologists independently without consensus reading, as each image was interpreted by a single reader in routine clinical practice. Although all readers have extensive experience in pediatric radiology and bone age assessment up to 30 years, the routine reports served as the reference standard rather than a true ground truth. Therefore, our study focused on the agreement between radiological reports and AI-based bone age assessment. Fourth, due to the software’s approved age range, children with a chronological age of < 3 years or ≥ 17 years could not be included in the present study. This represents an inherent limitation of the software, preventing its use and evaluation in these age groups. Furthermore, patients with forensic indications were excluded according to the manufacturer’s guidelines. In certain indication subgroups, the number of radiographs weakens statistical evaluation but reflects the real-world study requests.

## Conclusion

In conclusion, this study demonstrates a high level of agreement between the investigated AI algorithm and routine bone age assessments based on the G&P method in a large cohort of pediatric patients. Novel to previous studies, the indication for imaging was taken into account, revealing differences in agreement across underlying disease groups, with the highest agreement observed in patients with orthopedic indications. Given the use of routine radiology reports as the reference standard, these findings should be interpreted as reflecting agreement with expert clinical assessments rather than a definitive validation of algorithm accuracy.

## Supplementary information

ELECTRONIC SUPPLEMENTARY MATERIAL

## Data Availability

The datasets generated and analyzed during the current study are not publicly available but are available from the corresponding author on reasonable request

## Abbreviations

ICC: Intraclass correlation coefficient
MAD: Mean absolute difference
SD: Standard deviation
