# Supplementary material for: Beyond chronological age: uncovering disease-dependent variability in AI-driven pediatric bone age assessment
Source: Insights Imaging. 2026 Nov 14;17:294. doi: 10.1186/s13244-026-02477-0 (PMC13769785; doi:10.1186/s13244-026-02477-0)

Beyond Chronological Age: Uncovering Disease Dependent Variability in AI-driven Pediatric Bone Age Assessment

Electronic Supplementary Material

Supplemental Figures Bland-Altman plots demonstrate variable agreement between radiologist and AI-based bone age estimation among the different specific subgroups allocated to the three main indication groups (A genetic/neuroendocrine, B orthopedic and C oncologic) with best performances for the indications pubertas tarda and focal orthopedic conditions, and highest bias for the indication congenital adrenal hyperplasia. Continuous line: mean difference, dashed lines: upper and lower limits of agreement.

A Genetic/Neuroendocrine

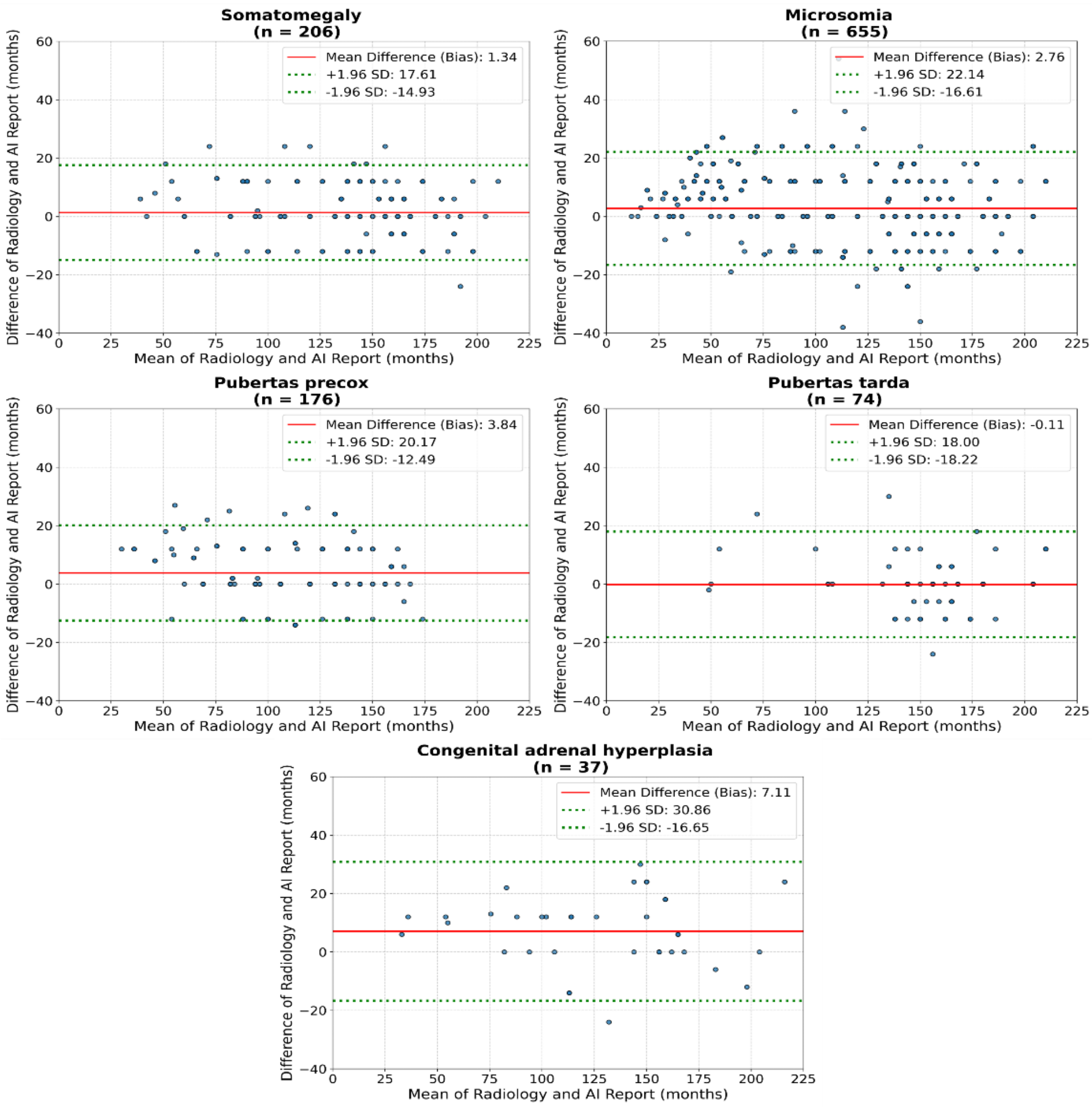

B Orthopedic

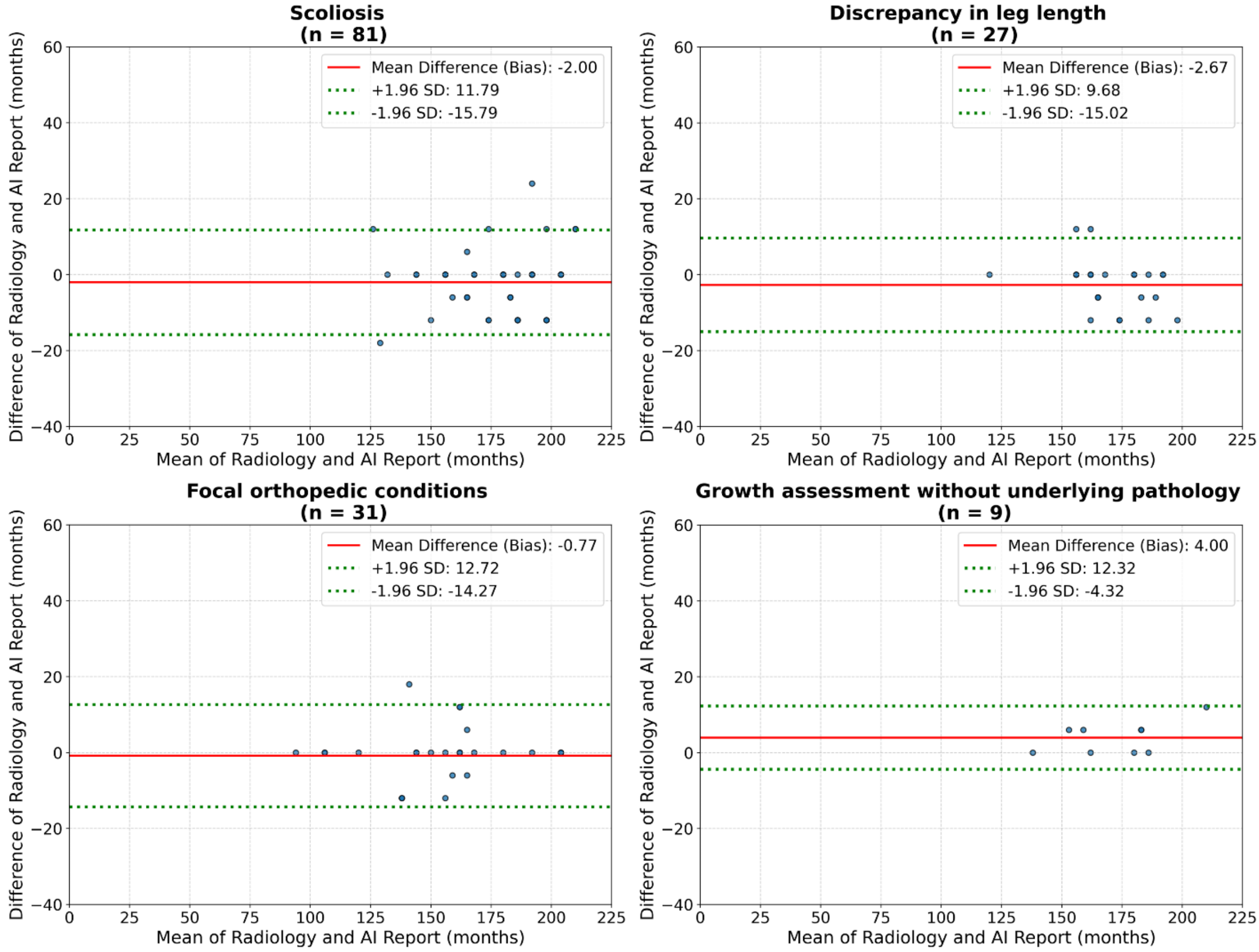

C Oncologic

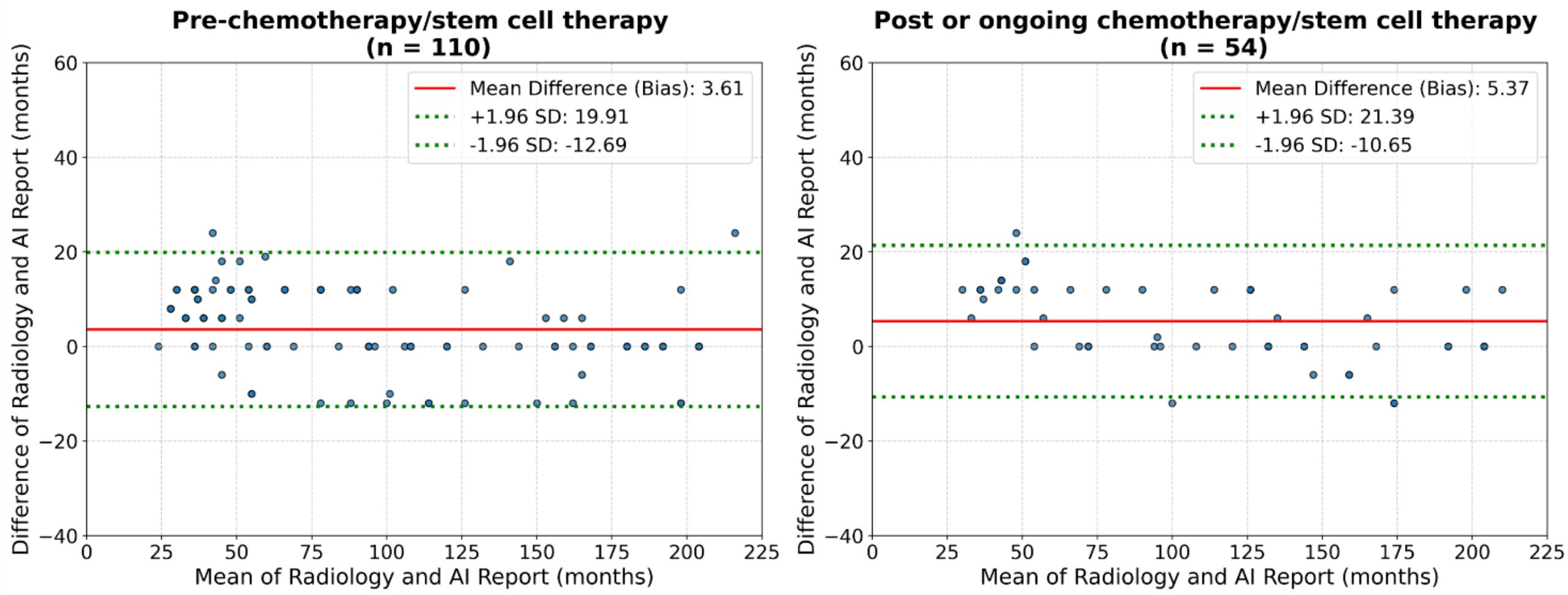

Supplement: Supplementary file 1 [file 13244_2026_2477_MOESM1_ESM.pdf]
